# Combination of Caloric Restriction and a Mixed Training Protocol as an Effective Strategy to Counteract the Deleterious Effects in Trabecular Bone Microarchitecture Caused by a Diet-Induced Obesity in Sprague Dawley Rats

**DOI:** 10.3390/nu14183672

**Published:** 2022-09-06

**Authors:** Elena Nebot, Rosario Martínez, Garyfallia Kapravelou, Cristina Sánchez, Juan Llopis, Pilar Aranda, Jesús M. Porres, María López-Jurado, Peter Pietschmann

**Affiliations:** 1 Department of Physiology, School of Medicine, Complutense University of Madrid, 28040 Madrid, Spain; 2 Department of Physiology, Institute of Nutrition and Food Technology (INyTA), Biomedical Research Center (CIBM), University of Granada, Avda del Conocimiento s/n, 18100 Armilla, Spain; 3 Institute of Pathophysiology and Allergy Research, Center for Pathophysiology, Infectiology and Immunology, Medical University of Vienna, 1090 Vienna, Austria

**Keywords:** bone microarchitecture, micro-CT, bone turnover markers, weight loss strategies, exercise, caloric restriction diet

## Abstract

The association of obesity with changes in bone mass is not clear. Obese individuals tend to have an increased bone mineral density, but other studies have shown that obesity is a major risk factor for fractures. The mechanisms of bone response during a weight loss therapy as well as the possible osteoprotective effect of exercise should be analyzed. The aim of this study was to test the effects of a weight-loss program based on the combination of caloric restriction and/or a mixed training protocol on different parameters of bone morphology and functionality in a DIO rat model. Three stages were established over a 21-week period (obesity induction 0–12 w, weight loss intervention 12–15 w, weight maintenance intervention 15–21 w) in 88 male Sprague Dawley rats. Bone microarchitecture, total mineral and elemental composition, and bone metabolism parameters were assessed. Weight loss interventions were associated to healthy changes in body composition, decreasing body fat and increasing lean body mass. On the other hand, obesity was related to a higher content of bone resorption and inflammatory markers, which was decreased by the weight control interventions. Caloric restriction led to marked changes in trabecular microarchitecture, with a significant decrease in total volume but no changes in bone volume (BV). In addition, the intervention diet caused an increase in trabeculae number and a decrease in trabecular spacing. The training protocol increased the pore diameter and reversed the changes in cortical porosity and density of BV induced by the high protein diet at diaphysis level. Regarding the weight-maintenance stage, diminished SMI values indicate the presence of more plate-like spongiosa in sedentary and exercise groups. In conclusion, the lifestyle interventions of caloric restriction and mixed training protocol implemented as weight loss strategies have been effective to counteract some of the deleterious effects caused by a dietary induction of obesity, specifically in trabecular bone morphometric parameters as well as bone mineral content.

## 1. Introduction

Overweight and obesity are defined as excessive and abnormal accumulation of fat that means a health risk. When combined with metabolic alterations such as hypertension, central obesity, insulin resistance, and/or dyslipidemia a cluster of pathologies shows up, denominated as metabolic syndrome (MetS). Due to their high prevalence in the world population, obesity and MetS are considered pandemics. Their incidence increases alarmingly every year mainly due to environmental factors, although genetic factors are also involved [1]. Regarding environmental factors, the regular intake of high fat and high fructose diets is directly related to the development of obesity, which is an important risk factor for the development of other associated chronic pathologies, such as insulin resistance, non-alcoholic fatty liver disease (NAFLD), as well as the alteration of bone functionality.

The development of obesity and MetS is favored by the consumption of unbalanced and hypercaloric diets. Therefore, the consumption of a balanced diet that provides adequate amounts of nutrients to treat or prevent these pathologies is highly recommended. Nevertheless, to implement a negative energy balance for weight-loss treatment, hypocaloric/hyper protein diets are usually prescribed. Moreover, increasing evidence on the beneficial effect of certain bioactive dietary components on both obesity and its comorbidities has been accumulating in recent years [2,3].

Related to lifestyle interventions, there are two core aspects to correct an altered energy balance: diet and physical exercise. The first step in the treatment of obesity is focused on losing extra weight and ameliorating the related metabolic alterations. Another important issue for patients who complete a weight loss program is to avoid the post-intervention rebound effect. The bodyweight regain usually takes place right after the end of weight loss intervention as weight loss programs are just transient [4]. A multidisciplinary approach is required, including lifestyle modifications [5] and, in some cases, the reinforcement with pharmacological treatment. On the other hand, physical activity plays an essential role in the prevention and treatment of obesity. It contributes to generating a negative energy balance, thus facilitating weight loss and avoiding the rebound effect and subsequent body weight regain [6]. It is well established that different training protocols induce changes in a variety of molecular mechanisms involved in numerous intracellular pathways related to glucose and lipid metabolism, inflammation, or antioxidant status [7]. The World Health Organization (WHO) recommends during adulthood at least 150–300 min of moderate-intensity aerobic physical activity; or at least 75–150 min of vigorous-intensity aerobic physical activity; or an equivalent combination of moderate- and vigorous-intensity activity throughout the week, for substantial health benefits [8]. Such exercise practice confers benefits for the following health outcomes: improved all-cause mortality, cardiovascular disease mortality, incident hypertension, site-specific cancers, and type-2 diabetes, as well as mental health (reduced symptoms of anxiety and depression); cognitive health, and sleep. In addition, adequate physical activity seems to be crucial for the proper development and maintenance of the skeleton, and it is necessary to clarify the effects of physical exercise combined with dietary interventions on structural parameters, histomorphometry, and bone metabolism.

The association of obesity with bone mass is contradictory; on the one hand, obese individuals tend to have an increased bone mineral density (BMD) mainly due to weight-induced loading of the bone [9]. On the other, many studies have shown that obesity is a major risk factor for fractures, and, especially, visceral adiposity is negatively associated with BMD and total mineral content in humans [10,11]. In rodent models, a high-fat diet (HFD) could massively affect bone health by reducing trabecular and/or cortical bone mass [12].

It has been reported that a weight-loss program induced by a caloric restrictive diet is linked to a concomitant accelerated bone loss. Studies conducted in obese women have found that such diets are associated with significant decreases in bone mass and total BMD, as well as an increased risk of fracture [13,14]. Moreover, it has been shown [15] that a moderate weight loss induced by a caloric restriction can increase bone resorption.

The association of a well-balanced diet with exercise is a key strategy to treat obesity. Regular exercise, known to induce beneficial effects on bone, could attenuate weight loss-induced bone loss. Nevertheless, the mechanisms of bone response during a weight loss therapy as well as the possible osteoprotective effect of exercise remain unclear [16].

Given the aforementioned, we hypothesized that our specific combined strategy of caloric restriction and physical exercise interventions could provide interesting benefits in the treatment of obesity and its related bone alterations. Thus, this study aimed to test the effects of a weight-loss program based on the combination of caloric restriction and/or a mixed training protocol on different parameters of bone morphology and functionality in a diet-induced obesity (DIO) model of Sprague Dawley rats.

## 2. Material and Methods

### 2.1. Animals, Diets, and Experimental Design

**Figure 1:**
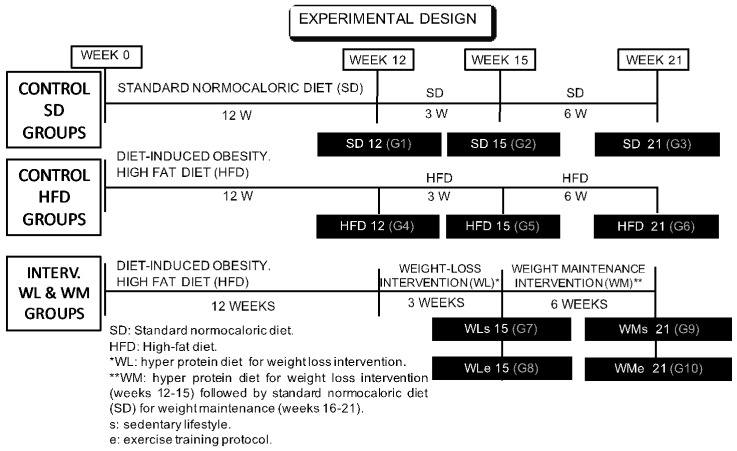
Experimental design: six control experiments were carried out for 21 weeks using a standard rat chow diet (Control SD groups: SD12, SD15 and SD21) or a high-fat diet to induce obesity (Control HFD groups: HFD12, HFD15 and HFD21). For intervention trials (Intervention WL & WM groups: WLs15, WLe15, WMs21, WMe21), rats were divided into 4 groups that were fed the hypercaloric diet to induce obesity for 12 weeks, continued by three weeks of intervention with a high-protein diet for weight loss (WL15), either following a sedentary lifestyle or combined with a training protocol (s or e, respectively). The intervention period was continued by an additional 6-week weight-maintenance stage of dietary treatment with a standard rat chow diet (WM21), either following a sedentary lifestyle or combined with a training protocol (s or e, respectively), in order to maintain the weight lost during the previous intervention period of three weeks.

The experiment used 88 male Sprague Dawley rats with an average body weight of 184 ± 10 g (6-weeks old, Charles Rives, Barcelona, Spain) that were allocated into eleven different experimental groups (*n* = 8). We only used male rats to avoid sex differences. The experiments lasted for 21 weeks and were divided into three stages (obesity induction and development of related alterations 0–12, weight loss intervention 12–15, weight maintenance intervention 15–21 weeks) (Figure 1).

Two control experiments that involved three groups of animals in each of them were organized with the following design:

1. Standard normocaloric groups (SD) fed a normocaloric standard rodent diet (Teklad Global Diet 2014; 2.4 Kcal/g) along the whole experiment:

G0. SD 0 weeks. Baseline control group.

G1. SD 12 weeks

G2. SD 15 weeks

G3. SD 21 weeks

2. Diet-induced obesity groups (HFD) fed a hypercaloric obesogenic diet containing 60% of Kcal as fat (Research diets D12492; 5.2 Kcal/g) along the whole experiment:

G4. HFD 12 weeks

G5. HFD 15 weeks

G6. HFD 21 weeks

In addition, four experimental groups were arranged with the following design:

3. Weight loss intervention groups (WL) based on caloric restriction and/or physical exercise during 3 weeks (weeks 13–15):

G7. A first stage for a period of 12 weeks consisting of dietary induction of obesity after ingestion of a hypercaloric diet (HFD), followed by a second 3-week stage (up to week 15) in which a caloric restriction dietary intervention to lose weight was implemented using an experimental diet designed to induce greater satiety combining the effects of high protein and soluble dietary fiber content (WLs 15) (2.9 Kcal/g). In addition to its satiating action, soluble dietary fiber is an effective therapeutic agent to treat many of the MetS components associated to obesity [17].

G8. Similar dietary intervention as in G7 complemented with a mixed training protocol implemented 5 days per week during weeks 13–15 of weight loss intervention (WLe 15).

For the following experimental groups, an additional third stage of the experiment ran for a period between weeks 16 to 21 and was designed for maintenance of lost body weight without any rebound effect:

G9. Similar dietary treatment as in G7 up to week 15 followed by ingestion of a SD diet for 6 weeks up to week 21 (WMs 21). The amount of food ingested during that 6-week period was *pair fed* to 23 g/d in order to achieve a 12–15% caloric reduction compared to the same period in control SD group as part of the strategy to maintain the lost weight during post-intervention stage avoiding the rebound effect.

G10. Similar dietary treatment as in G9 complemented during the intervention weight loss period (weeks 13–15) and weight-loss maintenance period (weeks 16–21) with a mixed training protocol implemented 5 days per week (Monday–Friday) (WMe 21).

The weight-loss intervention and body weight maintenance periods have been designed based on the information provided by Sengupta [18] who reported that laboratory rats live about 2–3.5 years (average 3 years), while the worldwide life expectancy of humans is 80 years. Thus, one human year almost equals two rat weeks (13.8 rat days) while correlating their entire life span. Under our experimental conditions, 3 weeks is equal to 1.5 years of human life, which is a long enough period to achieve an efficient weight loss. The maintenance period of 6 weeks is equal to 3 years, which is enough to demonstrate the success of our combined strategy against body weight regain.

The animals were housed in a well-ventilated, thermostatically controlled room (21 ± 2 °C) (Unidad de Experimentación Animal, CIC, Universidad de Granada). A reversed 12:12 light/dark cycle was implemented so the animals would perform the training protocol in darkness. Throughout the trial, animals had free access to type 2 water (resistivity 15 MΩ^−cm^) and consumed the diet *ad libitum,* with the exception of the intervention groups in the last stage of the experiment that were adapted to slightly lower food intake (23 g/d) compared to that of the normocaloric control during the same period (28 g/d) in order to keep a certain degree of caloric adaptation to avoid body weight rebound, as recommended by weight control programs [19]. The diet was provided for all four animals in each cage but the body weight control was registered individually. Caloric intake was recorded daily whereas body weight was measured once a week. At the end of each experimental period (obesity induction and development of related alterations 12th week, weight loss intervention 15th week, weight maintenance intervention 21th week), the animals were fasted for 8 h. Then, body composition was assessed with a whole-body composition analyzer based on magnetic resonance imaging (EchoMRI™; EchoMedical Systems, Houston 145 TX) prior to being anesthetized with ketamine (75 mg/kg body weight) and xylazine (10 mg/kg body weight) and euthanized by cannulation of the abdominal aorta. Blood was collected (with heparin as anticoagulant) and centrifuged at 3000 rpm for 15 min to separate the plasma, which was subsequently removed and frozen in liquid nitrogen and stored at −80 °C. Epydidimal and abdominal fat was extracted and weighted. The femur was extracted, weighted, measured, and immediately frozen in liquid nitrogen and stored at −80 °C until bone mass and microarchitectural analysis were conducted. Tibiae was also extracted, weighted, and measured. Bone marrow was extracted, frozen in liquid nitrogen, and stored at −80 °C for the determination of RANKL, interleukin 10, and leptin. All experiments were undertaken according to Directional Guides Related to Animal Housing and Care [20] and all procedures were approved by the Animal Experimentation Ethics Committee of the University of Granada, Spain (Project Reference DEP2014-58296R).

### 2.2. Training Protocol

Rats trained following a protocol based on interval aerobic training combined with strength exercise in the same session [21,22]. The animals ran on a specially designed treadmill (Panlab, LE 8710R) and all sessions were performed 5 days/week and during the dark cycle of the animals (active period). The training protocol was designed due to the beneficial effects of high-intensity interval aerobic training on obesity and parameters of lipid metabolism, and combined with an aerobic strength training protocol with effective action on insulin sensitivity and lipid profile. To establish the velocity that would correspond to the VO_2_ max of each rat, a maximal incremental test was performed at the start of the study. A final incremental test was performed 96 h prior the end of the study to test the maximal aerobic capacity and physical performance achieved by the animals as a result of the intervention. All sessions of the mixed training protocol consisted of 60 min of effective work. The sessions started with a 10-min warm-up at 35–50% maximal oxygen consumption (Supplementary Table S1), followed by the strength training consisting on eight 2-min running bouts separated by 1 min of rest during which animals ran with an inclination, progressively increased every three weeks from 10° up to 20° at a constant slow speed (20–25 cm/s, equivalent to 30–40% maximal oxygen consumption). The strength exercise was followed by 30 min of aerobic interval exercise, alternating 4 min bouts at 50–65% maximal oxygen consumption with 3 min bouts at submaximal intensity at 65–85% maximal oxygen consumption.

### 2.3. Bone Marrow Analyses

Bone marrow was extracted from tibiae by cutting lower portion of the bone and centrifuging at 6000 rpm. RANKL was measured with the rat kit Milliplex Rat RANKL (MAP kit, Millipore, Burlington, MA, USA), the cytokine interleukin (IL-1β) was measured with the rat kit Milliplex Rat Cytokine (MAP kit, Millipore), and leptin was measured with the rat kit Milliplex Rat Leptin (MAP kit, Millipore) and calibrated with Luminex 100/200 Calibration kit.

### 2.4. Assessment of Bone Mass and Bone Microarchitecture

Bone microarchitecture parameters of the femora were analyzed by μCT using a μCT-50 device (ScancoMedical, CH, Wangen-Brüttisellen, Switzerland). The long axis of the biopsies was oriented along the rotation axis of the scanner. The X-ray tube was operated at 70 kV with an intensity of 200 μA, and an exposure time of 500 ms, resulting in a resolution of 10 μm/pixel. Femora were scanned in a cortical region (mid-shaft and extending a 10% of the whole femur length) and in a trabecular region (proximal of the knee joint extending to a 10% at distal length of 75% of the whole femur length).

### 2.5. Ash Measurement and Elemental Composition of Femur

Femur samples were cleaned of flesh and debris before being weighed and their length measured. The samples were then freeze-dried, weighed, and processed for measurement of total mineral content after calcination in an oven at 450 °C for 5 days to a constant weight or processed for elemental analysis by wet digestion. The concentration of Mg, Ca, V, Mn, Fe, Co, Zn, As, and Se in femur was determined using inductively coupled plasma mass spectrometry (ICP-MS) following the protocol previously described by Sánchez-González et al. [23].

### 2.6. Statistical Analyses

For a more accurate description and interpretation of the data, the experimental period has been divided in three different stages: (i) dietary induction of obesity during weeks 0–12, (ii) individual or combined weight loss interventions: dietary (caloric restriction) and/or lifestyle (training protocol of mixed exercise during weeks 13–15, and (iii) post-intervention maintenance stage with normocaloric, diet combined or not with a training protocol weeks 16–21.

Significant differences in final body weight, body weight changes, caloric intake, body weight/tibial length ratio, body composition parameters, bone microarchitecture parameters and bone elemental composition were analyzed by *t*-test at 12 weeks of the experimental period, and by one-way ANOVA at 15 and 21 weeks of experimental period. Duncan’s test was used to detect differences between treatment means. Statistical analysis was performed with the Statistical Package for Social Sciences (IBM SPSS for Windows^®^, version 22.0, Armonk, NY, USA), and the level of significance was set at *p* < 0.05.

## 3. Results and Discussion

### 3.1. Caloric Intake, Body Weight Changes and Body Composition

**Figure 2:**
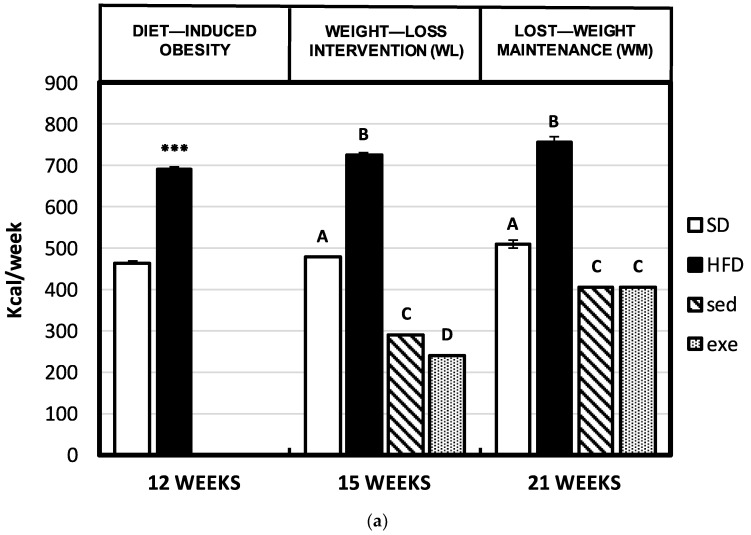

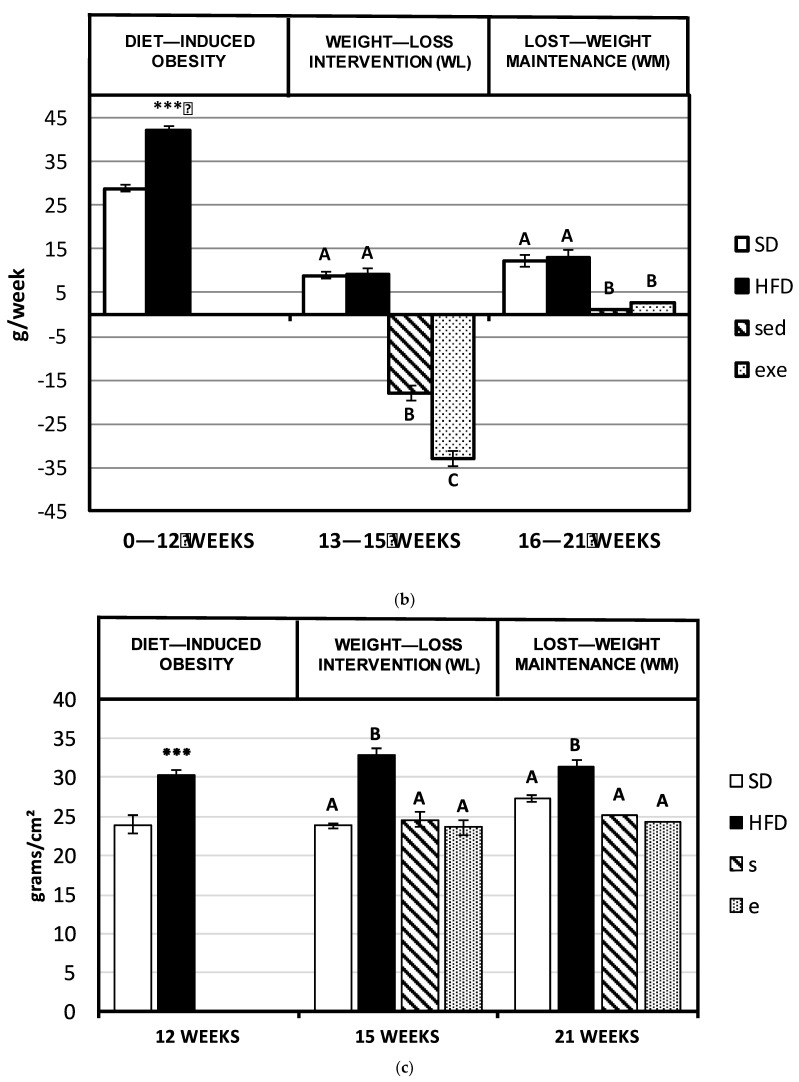
(**a**)Average weekly caloric intake (kcal/week) along the different experimental stages (DIO, weight-loss intervention, and weight-maintenance). Results are means of eight rats ± SEM depicted by vertical bars. *** *p* < 0.001 in *t*-test (12 weeks); A, B, C, D means with different letters are significantly different (ANOVA treatment, *p* < 0.05; 15 and 21 weeks). SD, standard normocaloric diet; HFD, high-fat diet; sed, sedentary lifestyle; exe, exercise training protocol. (**b**) Average weekly body weight changes (g/week) along the different experimental stages (DIO, weight-loss intervention, and weight-maintenance). Results are means of eight rats ± SEM depicted by vertical bars. *** *p* < 0.001 in *t*-test (12 weeks); A, B, C means with different letters are significantly different (ANOVA treatment, *p* < 0.05; 15 and 21 weeks). SD, standard normocaloric diet; HFD, high-fat diet; sed, sedentary lifestyle; exe, exercise training protocol. (**c**) Body weight/ tibial length ratio (g/cm^2^) at the end of the different experimental stages (DIO, weight-loss intervention, and weight-maintenance). Results are means of eight rats ± SEM depicted by vertical bars. *** *p* <0.001 in *t*-test (12 weeks); A, B means with different letters are significantly different (ANOVA treatment, *p* < 0.05; 15 and 21 weeks). SD, standard normocaloric diet; HFD, high-fat diet; sed, sedentary lifestyle; exe, exercise training protocol.

The effects of a high-fat diet intake (12 weeks) followed by an intervention high-protein diet (3 weeks) and weight maintenance normocaloric diet (6 weeks) combined or not with the training program on bodyweight change and body composition are shown in Figure 2a,b and in Table 1. As expected, caloric intake was significantly higher in the group fed the high fat diet compared to the group fed a standard normocaloric diet on weeks 12th, 15th and 21st, leading to higher body weight gain (expressed as g/week) only on week 12th. The treatment with high-protein diet during weeks 13–15 caused a significant weight loss, which was stronger when dietary treatment was combined with exercise.

During the weight maintenance stage (16–21 weeks), the intake of a normocaloric diet combined or not with exercise led to stabilization of body weight without any further gain or loss. Therefore, net body weight remained significantly lower in the intervention groups compared to both standard diet or high fat diet fed animals.

Regarding body composition, the significantly higher bodyweight found in obese groups (HFD 12, 15, and 21) was linked to similar lean body mass and total water but significantly higher total fat mass, abdominal fat and epididymal fat, than the groups fed the standard diet. The weight loss intervention (weeks 13–15) led to a significant reduction in weight (similar values were found in sedentary and exercised groups), whereas LBM was maintained, and fat mass was significantly reduced. Along the weeks 16–21 an effective maintenance of lost weight was achieved, preserving LBM levels with similar values to the group fed a standard diet (either in sedentary or exercised groups) and maintaining the low levels of total, abdominal and epididymal fat achieved in the previous stage. Exercise induced a further weight loss associated with an additional and significant loss of total, abdominal and epididymal fat, and increased the amount of LBM and the LBM to total body weight ratio on week 21. All these changes were reflected in the bodyweight to tibial length ratio that was significantly higher in obese vs. control normocaloric groups and returned to values similar to the normocaloric controls after the weight-loss and lost-weight maintenance interventions (Figure 2c).

#### Discussion

At the beginning of the weight loss intervention period obese rats nearly doubled the percentage body fat (18.4% to 10.6%) and were significantly heavier compared with SD rats of the same age. In contrast, the contribution of LBM to total weight was lower in those animals. Obese rats responded to caloric restriction (CR) with an efficient weight loss, mainly due to fat loss, while lean body mass increased in percentual terms. Bertrand et al. [24] found that this reduction in fat content is due to a lifelong decrease in both the size of individual adipocytes and the number of adipocytes in the fat depots. Barzilai and Gupta [25] have reported that CR is particularly effective in decreasing visceral fat in rats, in agreement to what has been reported in this experiment at the end of week 15, in which the percentage of abdominal fat in treated animals is half of that in obese rats not subjected to caloric restriction (3.5% to 6.1%). Besides, the exercise protocol followed enhanced the positive changes induced by CR intervention. Several investigations have demonstrated numerous adaptations to WAT in response to exercise that result in improved whole-body metabolic health [26]. These adaptations include increased mitochondrial biogenesis and gene expression [27,28,29,30] as well as changes in adipokine secretion. Certain beneficial effects of exercise may be mediated by an altered adipokine profile [31]. Among all the adipokines, leptin is significantly affected by exercise and acts as a satiety hormone to regulate energy balance through inhibition of hunger. The amount of circulating leptin correlates with adipose tissue mass, and a loss of adipose tissue mass in rodents and humans results in decreased serum concentrations of leptin [32,33], supporting the idea that adipose tissue plays an important role as a major endocrine organ that can be stimulated by exercise.

### 3.2. Bone Weight and Length, Metabolism Markers and Microarchitecture

The effects of obesity and weight-loss interventions on bone parameters are described in Table 2, Table 3 and Table 4. At the end of dietary induction of obesity stage (week 12), femur weight and length were higher in HFD vs. SD group. In contrast, no significant differences in tibial length or weight were observed between the former experimental groups. The intervention period with a high protein diet (13–15 w) lead to a decrease in femur weight compared to the HFD control, while a stabilization in femur length was observed in all experimental groups. The combination of exercise with a high protein diet did not induce any further changes in femur weight. During the weight maintenance period (16–21 w) femur weight results followed the same trend as in the previous stage, whereas exercise intervention tended to increase this parameter (Table 2).

Although due to high variability no statistically significant effects were established, a clear biological trend can be inferred to relate the development of obesity and increased content in medulla of the bone resorption marker RANKL, and the inflammatory markers IL-1β and leptin. In general, the weight control interventions exhibited a positive effect on such markers, returning them to levels similar to those of the SD controls.

The effects of DIO and weight control interventions on bone microarchitecture are presented in Table 3 and Table 4. Obesity produced a significant decrease in BV/TV index, connectivity density (Conn.D), trabecular number (Tb.N), and mean density TV compared to the SD fed group. In contrast, it increased Structure Model Index (SMI) and trabecular spacing (Tb. Sp). The main changes induced by the intake of HFD along 12 weeks on cortical bone microarchitecture were higher TV and BV values as well as a significant decrease in mean pore diameter and Ct.Po associated to higher density of bone volume. These effects were still observed at the end of 15 w in the group fed HFD compared to the group fed SD, although results were not significant.

The intake of a high protein diet during the weight loss intervention period (w13–15) led to marked changes in trabecular microarchitecture, with a significant decrease in total volume (TV) but no changes in bone volume (BV). Therefore, the BV/TV index remained similar to the group fed HFD and significantly lower than the control fed SD. The intervention diet caused a significant increase in trabecular number (Tr.N) and a decrease in trabecular spacing (Tr. Sp), while the trabecular thickness remained lower than the values of either the HFD or the SD groups. A trend to recover the mean density of TV was apparent. The main changes observed during the same period in cortical microarchitecture were a marked increase in Ct.Po that runs in parallel to lower mean density of BV. The training protocol significantly increased the pore diameter and reversed the changes in Ct. Po and density of BV induced by the high protein diet. However, no significant alterations were observed in both TV and BV, leading to no changes in the BV/TV index.

The effectiveness of the intervention period for maintaining the weight loss achieved in the previous stage was very high. Concerning trabecular microarchitecture, the increase in trabecular number compared to the HFD group was also clear, reaching similar values to that of the SD group. Moreover, the decrease in trabecular spacing achieved in the previous period remained, and a higher value for the density of BV was also observed that reached significantly higher values than the HFD group as a result of the training protocol. Related to cortical microarchitecture, no major changes in any of the measured parameters and indices were apparent among the different experimental groups at this stage except for a higher pore diameter in the femur of trained rats.

#### Discussion

To our knowledge, this is the first study that has investigated the effects of a high fat diet intake (12 weeks) followed by a weight-loss intervention high-protein diet (three weeks) and weight maintenance normocaloric diet (six weeks) combined or not with a training program on bone metabolism parameters and trabecular and cortical microarchitecture in the femur of male adult rats.

Obesity has been traditionally linked to greater bone mineral content that might be expected to protect the skeleton [34]. Nevertheless, animal studies have shown that a relationship exists between obesity and poor bone quality in diet-induced obese animals [35,36]. Because BMD only partially explains bone strength, we investigated the bone quality by means of its micro-architecture. It is well known that trabecular bone structure parameters could be affected in DIO [37,38]. In accordance with these findings, our results also demonstrated notable trabecular bone loss and microarchitecture deterioration in the HFD group, as evidenced by decreased BV/TV, Conn.D, Tb.N, and density of TV, as well as increased Tb.Sp. In this regard, a study observed similar aggravated results in the cancellous bone of rats fed HFD (58% fat of total kcal) after 16 weeks [39]. Gautam et al. [40] also showed marked deterioration at the trabecular region in mice after 10 weeks of HFD treatment (60% fat). However, in their study, HFD did not alter cortical bone mass. On the other hand, Li et al. [41] found reduced bone density, Tb.Th, and Tb.N in Wistar rats fed with HFD (40% fat) compared to the standard diet group at 10th week, while the BV/TV was not significantly affected. Nevertheless, Cao et al. [35] reported decreased cancellous but not cortical bone mass in tibia of mice fed a 45% HFD for 14 weeks. In our study, the main changes on cortical bone microarchitecture induced by HFD along 12 weeks were higher TV and BV, as well as a significant decrease in mean pore diameter and Ct.Po associated to higher density of bone volume. Our results are consistent with the study by Cao et al. [35] showing no significant effects of HFD on tibial cortical thickness. Other study [37] showed unaffected both trabecular and cortical thickness in the fourth lumbar vertebra (L4) in mice aged 31 weeks and fed high-fat chow (60% fat) for 24 weeks being coincident with our results. Differences in the diet composition, age or strain of the rodent, length of the study, and the site (femur, tibia, vertebra) could account for these discrepancies [35]. In this regard, the effects of obesity on bone quality are complex and appear to vary with several factors, including age, sex, and site [42]. Obesity has been linked to a site-specific increase in fracture risk [43]. This risk has been partially attributed to a decrease in mineral content, but the relationship between obesity and bone mineral density is incompletely understood. Animal models of obesity show varying bone responses to obesity, with some studies showing an increase in bone formation and others a decrease [44,45].

In the present study, weight-loss intervention led to marked changes in trabecular microarchitecture. We observed trabecular changes as a decreased TV, but not the BV, meaning that this therapy did not significantly modify the BV/TV index in comparison with HFD. In this sense, we also observed a trend to recover the density of TV. These data suggest that, at this time point, the weight-loss intervention (with or without exercise) does not vary the cancellous bone mass and density in the femur of male adult rats. Nevertheless, the intervention was effective at improving other trabecular parameters such an elevated Tb.N together with a diminished Tb.Sp, but we did not observe any particular effect of the training protocol. Interestingly, the thickness of trabeculae was significantly diminished, although a trend of some positive effect of the exercise is clear; nevertheless, it was not fully reversed to that of the SD group. Similarly, Gerbaix et al. [16] observed that a well-balanced diet alone failed to alter total and tibia bone mass and BMD in obese rats after 2 months. However, Tb.Th and bone volume density of metaphysis were decreased by the diet. The moderate intensity exercise performed significantly improved BMD possibly by inhibiting the bone resorption without any trabecular and cortical adaptation. It is known that exercise training added to a diet-induced weight loss can attenuate the weight loss-induced reduction in BMD and lean body mass in obese human [46,47]. Just as reduced loading of bone such as that experienced during weight loss induce dramatic decreases in BMD, high forces that are rapidly developed may increase bone density by increased loading. In this regard, jump exercise during hindlimb unloading protects against adverse changes in trabecular bone microarchitecture in young rats [48].

In our study, at diaphysis level, the weight-loss intervention during the same period led to no changes in both BV and TV, independently of the sedentary lifestyle or the training protocol, suggesting a non-altered cortical bone mass. This finding is associated with a higher density of BV in the femora of the WLe group. The training protocol seems to improve the density of BV reaching the values of the control SD and HFD groups, meaning a beneficial effect on bone cortical microarchitecture despite the non-beneficial effect of the weight-loss intervention. This effect is consistent with a marked increase in the mean pore diameter and Ct.Po. However, the training protocol reversed the detrimental changes in Ct. Po and density of BV induced by the high protein diet.

Regarding the weight-maintenance stage with SD dietary treatment (weeks 15–21), we clearly observed an effect of both, the diet and the training protocol on trabecular microarchitecture. Combination of the SD dietary therapy with exercise was effective on the BV/TV and on the density of TV, which were evidently increased in this group (WMe). Moreover, changes in the SMI were detectable in both groups (WMs and WMe). SMI describes if an examined volume of trabecular bone has either plate- or rod-like properties and is thus a suitable tool to describe subtle ongoing changes in bone microarchitecture. The values for mammalian spongiosa range from 0 to 3, with 0 being the ideal plate and 3 the ideal rod [49]. In our study, significantly decreased SMI values indicate the presence of more plate-like spongiosa in both groups. Interestingly, and in a different way to what happened in the weight-loss intervention, trabecular thickness remained relatively unaffected in all groups. This finding was already explained by Patsch et al. [37] considering the computation of the parameter itself: simplified, trabecular thickness is calculated as the most frequently occurring diameter of a virtual ball fitting into trabecular structures [50]. Furthermore, the increase in Tb.N compared to the HFD group, reaching similar values to that of the SD group, confirms the effectiveness of the weight-maintenance stage with SD dietary treatment. Also, the decrease in trabecular spacing achieved in the previous period remained. Related to cortical microarchitecture, the WM groups showed no significant alterations in any of the measured parameters and indices among the different experimental groups, but an exaggerated increase in the mean pore diameter in the femur of trained rats. Similar to our findings, Scheller et al. [12] investigated the impact of HFD (60%) and subsequent weight loss on skeletal parameters in six-week old male mice. They observed decreased trabecular bone volume fraction, mineral content, and number after 12, 16, or 20 weeks of HFD compared to normal chow diet controls, with only partial recovery after weight loss (HFD for 12 weeks and then normal chow for eight weeks to mimic weight loss).

In general terms, our study demonstrates that there are some reversible and some permanent changes in bone quality with HFD, followed by WL. Diet-induced obesity causes greater damage in growing bones [51]. Indeed, our animals started HFD at an age in which skeletal development is still highly active, likely contributing to impaired bone accrual during growth. For healthy aging, different regimens may be required to maintain bone health after WL, possibly with a focus on activity and diet. Training strategies that include heavy resistance training and high impact loading may be especially productive in maintaining, or even increasing bone density with weight loss [52].

### 3.3. Ash and Bone Mineral Content

The effects of DIO and bodyweight control interventions on the elemental composition of the femur are presented in Table 5. Although total mineral content represented by ash percentage did not differ significantly among all the groups at the different stages of the experimental period, some differences were apparent in specific elements. Generally, the dietary induction of obesity caused a decrease in femur content of macrominerals P, Ca, and Mg that was significant at week 12 of the experimental period but did not reach statistical significance at week 15. By week 21 such differences were not apparent. Obesity induction also caused a significant decrease in the content of certain microminerals and trace elements such as Cu, Mn, Co, or As that remained on weeks 15 and 21 except for Cu in the later stage. Besides, the femur content of K and Fe was significantly decreased by obesity from week 15 of the experimental period. The effects of weight control interventions were not consistent for all the minerals studied, but tended to reverse the previously described action of obesity in P, Ca, Mg, Fe and Mn.

#### Discussion

Metabolic syndrome and osteoporosis have been described to share some common underlying pathways, such as regulation of calcium homeostasis, receptor activator of NF-κB ligand (RANKL)/receptor activator of the NF-κB (RANK)/osteoprotegerin (OPG), and Wnt-β-catenin signaling pathways [53]. Thus, metabolic syndrome may have a potential role in the development of osteoporosis. In this regard, mineral homeostasis is significantly affected in murine DIO models. The content of several elements in the liver, kidney, heart, and pancreas has been shown to decrease in response to high-fat diet feeding [54]. Such altered trace elements status is supposed to be a primary modification and precedes other metabolic obesity-related disturbances. Nevertheless, treatment of obesity-related symptoms alleviated the altered trace elements metabolism induced by HFD by modulating hyperglycemic and insulin resistance status [55]. Likewise, altered renal functionality derived from T2DM, one of the associated metabolic disturbances of obesity in metabolic syndrome, has been described to affect mineral metabolism and modify the elemental composition of plasma and bone [56]. Here, the development of obesity appeared to exert an inhibitory action on the bone mineral content of both macro and micro or trace elements, although no differences in total mineral content of bone were observed despite morphometric changes associated with obesity. In this regard, Ip et al. [57] reported no significant differences in femur ash content of obese Zucker rats vs. their lean counterparts although a smaller femur size and weight was apparent in the obese animals. On the other hand, Song and Sergeev [58] found a significant decrease in femur Ca and P content of HFD-treated mice. Such deleterious effects were reversed by high intakes of vitamin D-3 and calcium.

Modifications of femur mineral content in obese animals was correlated to the changes in microarchitecture parameters observed in trabecular rather than cortical bone. In the former, a significant decrease in bone volume, connectivity density, trabecular number, and BMD of total volume were caused by obesity. In addition to its possible relationship to microarchitecture changes, the lower concentration of minerals in the femur could also be due to a dilution effect caused by the accumulation of fat in the bone marrow.

Mathey et al. [59] eported that a continuous aerobic training protocol (35–40% VO_2max_, 20–50 min/day, 6 days/week, 89 days) induced an exercise-induced increment in BMD, bone calcium content, diameter, and femoral failure load. The mixed training protocol implemented in our experiment showed a trend to increase P, Ca, and Mg content when compared to their sedentary controls. Nevertheless, such an effect was observable on week 15 but not on week 21 of the experimental period. Other authors [60] have pointed out the beneficial effects on fructose-induced obese rats of exercise (1-h running protocol a day, six days per week, ten weeks) that reduced visceral fat and ameliorated glucose intolerance, lowered blood lactic acid levels, improved lactic acid usage efficiency, and increased oxidative stress and hepatic levels of Mn, Fe, Cu, and Zn in the normal and obese animals.

## 4. Conclusions

The lifestyle interventions of caloric restriction and mixed training protocol implemented as weight loss strategies have been effective to counteract some of the deleterious effects caused by dietary induction of obesity in a Sprague Dawley rat model, specifically in trabecular bone morphometric parameters and indices as well as on bone mineral content. Thus, the interventions can be used as efficient strategies in the treatment of obesity, although some modifications in the training protocol could be of interest to maintain or even increase bone density with weight loss.

## Figures and Tables

**Table 1:** Effects of obesity induction and weight control interventions on bodyweight and body composition.

	0 WEEKS (Baseline)	Diet-Induced Obesity 12 WEEKS		Weight-Loss Intervention (WL) 15 WEEKS				Lost-Weight Maintenance Intervention (WM) 21 WEEKS			
		SD	HFD	SD	HFD	WLs	WLe	SD	HFD	WMs	WMe
**Body weight (g)**	172.7 (2.21)	516.5 (18.9)	664.3 *** (14.1)	503 a (8.44)	705 b (15.1)	566 c (11.2)	552 abc (23.4)	631 a (19.8)	742 b (24.6)	639 a (9.33)	574 a (19.9)
**Lean body mass (g)**	155.9 (2.13)	422.2 (12.9)	453.4 (14.8)	414.3 a (8.21)	479 b (5.80)	448.2 ab (11.4)	442 ab (16.9)	481.8 a (12.9)	500.4 a (14.6)	490.3 a (9.67)	483.8 a (11.2)
**Total water (g)**	136.3 (1.92)	351.8 (10.7)	377.7 (9.64)	339.3 a (7.69)	397.6 b (5.78)	375.8 ab (11.8)	372.6 ab (13.1)	405.0 a (12.0)	416.1 a (24.4)	408.6 a (5.77)	409.1 a (11.4)
**ΔLBM/ΔBW**		0.78 (0.02)	0.61 *** (0.02)	0.76 a (0.02)	0.61 b (0.02)	0.74 a (0.02)	0.76 a (0.03)	0.71 a (0.03)	0.60 b (0.02)	0.74 ac (0.03)	0.82 c (0.03)
**LB/TW**	1.14 (0.004)	1.20 (0.02)	1.20 (0.02)	1.221 (0.02)	1.205 (0.02)	1.193 (0.02)	1.186 (0.02)	1.190 (0.02)	1.203 (0.02)	1.200 (0.02)	1.183 (0.02)
**Fat mass (g)**	8.06 (0.75)	54.8 (5.07)	122.8 *** (9.34)	49.2 a (6.68)	178.7 b (12.6)	73.3 a (10.2)	63.5 a (10.8)	90.6 a (7.54)	161.7 b (15.1)	110.7 a (8.70)	39.7 c (7.25)
**Abdominal fat (g)**	0.69 (0.07)	12.9 (1.14)	28.8 *** (2.16)	11.2 a (1.46)	43.0 b (3.0)	18.8 a (2.65)	16.2 a (2.32)	19.7 a (2.02)	38.5 b (2.49)	27.2 a (2.44)	9.88 c (1.57)
**Epydidimal fat (g)**	1.05 (0.09)	8.73 (0.79)	16.7 *** (0.88)	8.19 a (0.74)	20.7 b (1.10)	12.8 a (1.57)	12.2 a (1.72)	11.6 a (0.75)	21.0 b (1.61)	12.5 a (0.68)	7.34 c (0.61)

SD, standard rat chow diet; HFD, hypercaloric diet for dietary induction of obesity; WLs, high-protein weight-loss intervention diet with sedentary lifestyle (weeks 12–15); WLe, high-protein weight-loss intervention diet with training protocol; WMs, high-protein weight-loss intervention diet with sedentary lifestyle (weeks 12–15) followed by weight-maintenance stage (weeks 15–21) with SD dietary treatment and sedentary lifestyle; WMe, high-protein weight-loss intervention diet with training protocol followed by weight-maintenance stage with SD dietary treatment and training protocol. ΔLBM/ΔBW, changes in lean body mass vs. changes in body weight with respect to control baseline group; LB/TW, lean body mass vs. total water ratio at each experimental stage. Results are expressed as means of 8 rats and standard error of the mean (in parenthesis). *** *p* < 0.001 in *t*-test (12 weeks); a,b,c, means within the same line of each experimental stage (15 and 21 weeks) with different letters are significantly different (ANOVA treatment, *p* < 0.05).

**Table 2:** Effects of obesity and weight control interventions on bone anthropometry and bone markers of structure and functionality in bone marrow.

	0 WEEKS (Baseline)	Diet-Induced Obesity 12 WEEKS		Weight-Loss Intervention (WL) 15 WEEKS				Lost-Weight Maintenance Intervention (WM) 21 WEEKS			
		SD	HFD	SD	HFD	WLs	WLe	SD	HFD	WMs	WMe
**Femur weight (g)**	0.58 (0.02)	1.37 (0.04)	1.94 *** (0.09)	1.47 a (0.02)	1.82 b (0.03)	1.41 a (0.06)	1.40 a (0.06)	1.74 ab (0.04)	1.77 b (0.09)	1.57 a (0.03)	1.73 ab (0.06)
**Femur length (cm)**	2.89 (0.02)	4.15 (0.03)	4.35 ** (0.05)	4.21 a (0.04)	4.32 a (0.03)	4.28 a (0.05)	4.33 a (0.04)	4.36 a (0.04)	4.34 a (0.07)	4.36 a (0.04)	4.36 a (0.04)
**Tibial weight (g)**	0.74 (0.03)	1.44 (0.03)	1.54 (0.02)	1.31 (0.05)	1.56 (0.06)	1.58 (0.04)	1.52 (0.06)	1.57 (0.06)	1.72 (0.05)	1.53 (0.02)	1.68 (0.11)
**Tibial length (cm)**	3.36 (0.04)	4.66 (0.03)	4.64 (0.05)	4.59 (0.03)	4.65 (0.03)	4.79 (0.05)	4.77 (0.04)	4.82 (0.06)	4.92 (0.08)	4.73 (0.02)	4.84 (0.05)
**Bone marrow**											
**RANKL (pg/mL)**	1339.5 (216.9)	990.3 (87.6)	1624.5 (310.6)	1154.9 a (260.0)	1621.5 a (233.5)	916.7 a (209.4)	1044.5 a (179.5)	1021.6 a (280.3)	848.9 a (137.5)	1024 a (96.2)	1032.3 a (260.4)
**IL-1β (pg/mL)**	69.1 (7.12)	133.6 (11.6)	117.8 (7.38)	128.1 a (13.6)	139.9 a (25.7)	153.2 a (15.3)	126.0 a (6.70)	134.0 a (13.1)	180.6 a (46.9)	151.0 a (13.2)	159.7 a (13.7)
**Leptin (pg/mL)**	5.29 (1.17)	32.4 (7.41)	27.4 (9.20)	26.5 a (11.8)	46.2 a (15.3)	23.5 a (8.45)	35.2 a (10.2)	39.9 a (9.1)	59.9 a (19.2)	44.5 a (7.84)	34.4 a (13.1)

SD, standard rat chow diet; HFD, hypercaloric diet for dietary induction of obesity; WLs, high-protein weight-loss intervention diet with sedentary lifestyle (weeks 12–15); WLe, high-protein weight-loss intervention diet with training protocol; WMs, high-protein weight-loss intervention diet with sedentary lifestyle (weeks 12–15) followed by weight-maintenance stage (weeks 15–21) with SD dietary treatment and sedentary lifestyle; WMe, high-protein weight-loss intervention diet with training protocol followed by weight-maintenance stage with SD dietary treatment and training protocol. Results are expressed as means of 8 rats and standard error of the mean (in parenthesis). ** *p* < 0.01, *** *p* < 0.001 in *t*-test (12 weeks); a,b, means within the same line of each experimental stage (15 and 21 weeks) with different letters are significantly different (ANOVA treatment, *p* < 0.05). RANKL, Receptor Activator for Nuclear Factor κB Ligand.

**Table 3:** Effects of obesity and weight control interventions on 3D outcomes for trabecular (metaphysis) bone microarchitecture in femur.

	0 WEEKS (Baseline)	Diet-Induced Obesity 12 WEEKS		Weight-Loss Intervention (WL) 15 WEEKS				Lost-Weight Maintenance Intervention (WM) 21 WEEKS			
		SD	HFD	SD	HFD	WLs	WLe	SD	HFD	WMs	WMe
**TV (mm^3^)**	29.9 (0.9)	68.4 (3.3)	78.2 (4.0)	70.4 (2.5) ab	76.5 (1.9) b	65.1 (3.6) a	69.9 (2.9) ab	81.7 (3.2) b	76.6 (3.5) ab	70.9 (1.5) a	71.3 (2.7) a
**BV (mm^3^)**	2.78 (0.3)	20.8 (1.6)	18.6 (1.9)	22.4 (1.4) b	13.6 (0.9) a	12.7 (1.2) a	14.3 (1.1) a	19.4 (1.3) b	14.5 (0.6) a	17.8 (1.7) ab	19.1 (2.2) ab
**BV/TV**	0.09 (0.01)	0.30 (0.02)	0.24 * (0.02)	0.32 (0.02) b	0.18 (0.01) a	0.20 (0.02) a	0.21 (0.01) a	0.24 (0.02) ab	0.19 (0.01) a	0.25 (0.02) a	0.27 (0.03) b
**Conn. D (1/mm^3^)**	44.3 (10.4)	92.9 (4.1)	67.0 *** (3.5)	92.7 (4.9) b	52.9 (5.5) a	47.9 (2.3) a	48.2 (2.7) a	60.9 (3.1) b	45.5 (3.2) a	44.7 (4.2) a	44.8 (5.4) a
**SMI**	3.13 (0.1)	1.18 (0.2)	1.57 (0.1)	1.04 (0.1) a	1.91 (0.1) b	1.77 (0.1) b	1.69 (0.1) b	1.45 (0.1) bc	1.77 (0.1) c	1.19 (0.1) ab	0.98 (0.2) a
**Tb. N (1/mm)**	3.31 (0.23)	4.06 (0.13)	2.54 *** (0.28)	3.91 (0.13) c	2.08 (0.18) a	2.62 (0.12) b	2.64 (0.11) b	2.72 (0.23) b	2.06 (0.17) a	2.85 (0.18) b	2.84 (0.21) b
**Tb. Th (mm)**	0.051 (0.002)	0.096 (0.004)	0.099 (0.004)	0.101 (0.005) c	0.091 (0.002) b	0.074 (0.003) a	0.077 (0.002) a	0.098 (0.005) b	0.093 (0.003) ab	0.087 (0.002) a	0.093 (0.003) ab
**Tb. Sp (mm)**	0.32 (0.02)	0.24 (0.01)	0.45 ** (0.06)	0.25 (0.01) a	0.53 (0.05) b	0.31 (0.02) a	0.31 (0.02) a	0.40 (0.04) b	0.53 (0.04) c	0.27 (0.03) a	0.27 (0.03) a
**Mean density TV (mg HA/cm^3^)**	143.9 (11.4)	295.4 (16.9)	232.2 * (16.8)	302.7 (12.8) b	177.8 (11.3) a	202.1 (11.9) a	208.4 (11.7) a	238.5 (18.5) ab	196.7 (10.4) a	237.8 (16.4) ab	253.6 (16.9)b
**Mean density BV (mg HA/cm^3^)**	645.9 (2.9)	787.9 (6.0)	802.1 (6.9)	795.1 (7.6) a	791.8 (7.2) a	794.3 (5.0) a	792.1 (7.0) a	807.0 (8.6) a	811.4 (6.6) a	810.5 (5.1) a	817.8 (4.6) a

SD, standard rat chow diet; HFD, hypercaloric diet for dietary induction of obesity; WLs, high-protein weight-loss intervention diet with sedentary lifestyle (weeks 12–15); WLe, high-protein weight-loss intervention diet with training protocol; WMs, high-protein weight-loss intervention diet with sedentary lifestyle (weeks 12–15) followed by weight-maintenance stage (weeks 15–21) with SD dietary treatment and sedentary lifestyle; WMe, high-protein weight-loss intervention diet with training protocol followed by weight-maintenance stage with SD dietary treatment and training protocol. Results are expressed as means of 8 rats and standard error of the mean (in parenthesis). * *p* < 0.05, ** *p* < 0.01, *** *p* < 0.001 in *t*-test (12 weeks); a,b,c, means within the same line of each experimental stage (15 and 21 weeks) with different letters are significantly different (ANOVA treatment, *p* < 0.05). TV, total volume, BV, bone volume, BV/TV, bone volume density, Conn. D, connectivity density, SMI, Structure Model Index, Tb. N, trabecular number, Tb. Th, trabecular thickness, Tb. Sp, trabecular spacing, HA, hydroxyapatite.

**Table 4:** Effects of obesity and weight control interventions on 3D outcomes for cortical (diaphysis) bone microarchitecture in femur.

	0 WEEKS (Baseline)	Diet-Induced Obesity 12 WEEKS		Weight-Loss Intervention (WL) 15 WEEKS				Lost-Weight Maintenance Intervention (WM) 21 WEEKS			
		SD	HFD	SD	HFD	WLs	WLe	SD	HFD	WMs	WMe
**TV (mm^3^)**	22.0 (0.4)	56.4 (2.8)	64.2 (3.5)	56.7 (1.36) a	64.1 (1.43) b	60.1 (2.80) b	62.9 (2.38) b	69.8 (2.1) a	69.7 (3.2) a	70.1 (2.5) a	71.5 (3.0) a
**BV (mm^3^)**	9.26 (0.15)	33.8 (1.3)	39.3 (0.04) *	35.1 (0.82) a	39.9 (1.18) b	38.0 (1.58) b	39.4 (1.82) b	43.6 (0.9) a	44.2 (2.1) a	44.8 (1.3) a	45.6 (1.8) a
**BV/TV**	0.42 (0.09)	0.60 (0.01)	0.61 (0.003)	0.62 (0.01) a	0.62 (0.01) a	0.63 (0.01) a	0.63 (0.01) a	0.63 (0.01) a	0.63 (0.01) a	0.64 (0.01) a	0.64 (0.01) a
**Ct. Th (mm)**	0.35 (0.01)	0.77 (0.02)	0.78 (0.06)	0.80 (0.02) a	0.84 (0.02) a	0.85 (0.02) a	0.85 (0.02) a	0.87 (0.02) a	0.89 (0.02) a	0.90 (0.02) a	0.91 (0.03) a
**Mean pore diameter (mm)**	0.063 (0.002)	0.040 (0.005)	0.028 (0.003) *	0.047 (0.007) ab	0.035 (0.007) a	0.050 (0.009) ab	0.085 (0.02) b	0.038 (0.006) ab	0.031 (0.004) a	0.042 (0.008) ab	0.087 (0.02) b
**Ct.Po (%)**	0.148 (0.004)	0.049 (0.001)	0.041 (0.001) ***	0.044 (0.001) a	0.041 (0.001) a	0.050 (0.001) b	0.044 (0.002) a	0.038 (0.001) a	0.037 (0.001) a	0.043 (0.004) a	0.039 (0.002) a
**Mean density of BV (mg HA/cm^3^)**	897.6 (4.8)	1050.6 (4.6)	1081.4 (4.9) ***	1074.3 (2.8) b	1083.9 (3.9) b	1022.8 (5.32) a	1068.2 (11.8) b	1107.2 (4.7) a	1098.5 (5.0) a	1088.9 (14.5) a	1101.5 (7.9) a

SD, standard rat chow diet; HFD, hypercaloric diet for dietary induction of obesity; WLs, high-protein weight-loss intervention diet with sedentary lifestyle (weeks 12–15); WLe, high-protein weight-loss intervention diet with training protocol; WMs, high-protein weight-loss intervention diet with sedentary lifestyle (weeks 12–15) followed by weight-maintenance stage (weeks 15–21) with SD dietary treatment and sedentary lifestyle; WMe, high-protein weight-loss intervention diet with training protocol followed by weight-maintenance stage with SD dietary treatment and training protocol. Results are expressed as means of 8 rats and standard error of the mean (in parenthesis). * *p* < 0.05, *** *p* < 0.001 in *t*-test (12 weeks); a,b, means within the same line of each experimental stage (15 and 21 weeks) with different letters are significantly different (ANOVA treatment, *p* < 0.05). TV, total volume, BV, bone volume, BV/TV, bone volume density, Ct. Th, cortical thickness, Ct.Po, cortical porosity, HA, hydroxyapatite.

**Table 5:** Influence of obesity and weight control interventions on mineral content of femur.

	0 WEEKS (Baseline)	Diet-Induced Obesity 12 WEEKS		Weight-Loss Intervention (WL) 15 WEEKS				Lost-Weight Maintenance Intervention (WM) 21 WEEKS			
		SD	HFD	SD	HFD	WLs	WLe	SD	HFD	WMs	WMe
**Ash (%)**	53.3 (0.9)	58.3 (1.1)	57.4 (1.5)	56.2 a (0.4)	56.7 a (0.9)	60.7 a (1.4)	58.7 ab (0.8)	58.4 a (0.7)	58.5 a (0.9)	59.5 a (0.7)	58.2 a (1.4)
**P (g/kg)**	94.2 (2.3)	129.6 (3.8)	107.3 *** (3.1)	116.6 a (5.1)	107.8 a (3.0)	114.3 a (5.3)	132.0 a (3.4)	119.6 a (3.1)	131.3 b (4.1)	130.6 b (1.7)	117.9 a (2.8)
**Ca (g/kg)**	177.9 (4.7)	255.8 (7.3)	225.7 * (6.7)	231.6 a (9.4)	224.3 a (6.3)	228.8 a (1.2)	254.2 a (3.8)	247.3 ab (6.6)	237.5 a (7.2)	259.5 b (3.2)	237.1 a (5.5)
**Mg (g/kg)**	3.42 (0.08)	4.39 (0.15)	3.13 *** (0.12)	3.54 ab (0.20)	3.06 a (0.10)	3.35 ab (0.16)	4.17 b (0.17)	3.72 b (0.13)	4.17 c (0.15)	3.78 b (0.06)	3.36 a (0.11)
**K (g/kg)**	5.04 (0.14)	1.71 (0.07)	1.79 (0.16)	2.36 c (0.20)	1.82 ab (0.12)	1.95 b (0.10)	1.51 a (0.07)	1.62 a (0.10)	1.74 a (0.08)	1.58 a (0.08)	1.76 a (0.12)
**Fe (mg/kg)**	88.5 (6.4)	45.2 (7.1)	44.0 (7.8)	68.6 ab (6.7)	46.8 a (4.3)	72.1 ab (9.1)	84.5 b (17.4)	51.0 a (3.5)	69.4 a (8.4)	70.8 a (11.4)	66.5 a (7.7)
**Zn (mg/kg)**	221.3 (11.5)	219.1 (6.6)	216.8 (10.8)	200.4 a (6.9)	215.8 a (3.5)	244.9 b (10.4)	241.5 b (8.7)	186.2 a (7.2)	235.8 bc (7.3)	257.5 c (6.8)	230.8 b (4.5)
**Cu (mg/kg)**	2.11 (0.22)	1.00 (0.17)	0.78 * (0.05)	1.57 b (0.08)	0.91 a (0.05)	1.93 c (0.06)	0.82 a (0.05)	0.73 a (0.04)	1.01 ab (0.05)	1.24 bc (0.06)	1.63 c (0.08)
**Mn (µg/kg)**	428.1 (16.3)	358.1 (86.7)	205.0 *** (21.5)	326.2 ab (43.4)	189.8 a (15.2)	374.9 b (22.8)	288.2 ab (28.3)	219.3 a (17.4)	186.4 a (9.0)	401.9 c (32.5)	309.8 b (11.7)
**Se (µg/kg)**	395.2 (18.8)	238.8 (18.2)	245.8 (14.3)	272.9 a (14.3)	261.0 a (14.8)	229.8 a (26.4)	274.6 a (22.1)	244.5 a (20.3)	266.6 a (20.5)	218.6 a (17.5)	239.8 a (14.2)
**V (µg/kg)**	10.7 (1.0)	31.0 (6.3)	19.5 (6.7)	15.4 a (4.4)	60.4 c (6.7)	34.1 b (3.8)	26.4 ab (4.2)	48.9 ab (6.4)	61.7 b (4.9)	26.5 a (2.1)	25.1 a (2.4)
**Co (µg/kg)**	56.9 (2.2)	76.5 (2.3)	62.8 *** (2.0)	79.1 bc (4.1)	67.4 ab (2.6)	89.0 c (5.6)	62.3 a (1.4)	88.5 d (2.9)	51.8 a (3.8)	79.0 c (2.9)	69.4 b (1.8)
**Sc (µg/kg)**	435.8 (13.5)	483.8 (37.6)	551.9 (15.0)	509.3 ab (29.0)	565.5 bc (16.4)	612.7 c (27.0)	442.4 a (13.5)	631.3 b (17.6)	666.4 b (22.2)	502.0 a (8.0)	493.3 a (13.6)
**As (µg/kg)**	67.8 (4.5)	91.2 (11.4)	23.9 *** (2.7)	84.5 c (5.3)	24.6 a (1.8)	40.5 b (5.1)	52.0 b (4.9)	75.3 c (4.8)	40.9 a (3.1)	58.0 b (5.2)	58.2 b (7.0)

SD, standard rat chow diet; HFD, hypercaloric diet for dietary induction of obesity; WLs, high-protein weight-loss intervention diet with sedentary lifestyle (weeks 12–15); WLe, high-protein weight-loss intervention diet with training protocol; WMs, high-protein weight-loss intervention diet with sedentary lifestyle (weeks 12–15) followed by weight-maintenance stage (weeks 15–21) with SD dietary treatment and sedentary lifestyle; WMe, high-protein weight-loss intervention diet with training protocol followed by weight-maintenance stage with SD dietary treatment and training protocol. Results are expressed as means of 8 rats and standard error of the mean (in parenthesis). * *p* < 0.05, *** *p* < 0.001 in *t*-test (12 weeks); a,b,c, means within the same line of each experimental stage (15 and 21 weeks) with different letters are significantly different (ANOVA treatment, *p* < 0.05).

## Data Availability

Not applicable.

## Supplementary Materials

The following supporting information can be downloaded at: https://www.mdpi.com/article/10.3390/nu14183672/s1, Table S1: Details of the mixed training protocol*.
