# Supplementary material for: Combination of Caloric Restriction and a Mixed Training Protocol as an Effective Strategy to Counteract the Deleterious Effects in Trabecular Bone Microarchitecture Caused by a Diet-Induced Obesity in Sprague Dawley Rats
Source: Nutrients. 2022 Sep 6;14(18):3672. doi: 10.3390/nu14183672 (PMC9504808; doi:10.3390/nu14183672)
Supplement: Supplementary file 1 [file nutrients-14-03672-s001.zip › nutrients-1846643-supplementary.pdf]

Suppl. Table S1. Details of the mixed training protocol*.

		Week 1	Week 2	Week 3	Week 4	Week 5	Week 6	Week 7	Week 8
**Warm up (10’)**		35% VO_2max	35% VO_2max	40% VO_2max	40% VO_2max	45% VO_2max	45% VO_2max	50% VO_2max	50% VO_2max
**Strength training** Eight 2-min running bouts separated by 1 min of rest	**Slope**	10%	10%	15%	15%	15%	20%	20%	20%
	Speed	20cm/s	25cm/s	20cm/s	23cm/s	26cm/s	20cm/s	23cm/s	26cm/s
**Aerobic Interval Training** (30’)	**Work Load**	4’ 50% VO_2max 3’ 60% VO_2max	4’ 50% VO_2max 3’ 65% VO_2max	4’ 55% VO_2max- 3’ 65% VO_2max	4’ 55% VO_2max 3’ 70% VO_2max	4’ 60% VO_2max 3’ 70% VO_2max	4’ 60% VO_2max 3’ 70% VO_2max	4’ 65% VO_2max 3’ 75% VO_2max	4’ 65% VO_2max 3’ 75% VO_2max

* Taken from Martínez, R., et al., Effects of a combined intervention with a lentil protein hydrolysate and a mixed training protocol on the lipid metabolism and hepatic markers of NAFLD in Zucker rats. *Food Funct*, **2018**. *9*(2): 830-850.
